# Editorial: Health and well-being, quality education, gender equality, decent work and inequalities: the contribution of psychology in achieving the objectives of the Agenda 2030

**DOI:** 10.3389/fpsyg.2024.1353102

**Published:** 2024-01-29

**Authors:** Andrea Zammitti, Celia Moreno-Morilla, Soledad Romero-Rodríguez, Paola Magnano, Jenny Marcionetti, Anna Parola

**Affiliations:** ^1^Department of Educational Sciences, University of Catania, Catania, Italy; ^2^Department of Didactics and Educational Organization, Faculty of Education Sciences, University of Seville, Seville, Spain; ^3^Department of Research and Assessment Methods in Education, School of Education, University of Seville, Seville, Spain; ^4^Department of Human and Social Sciences, Kore University of Enna, Enna, Italy; ^5^Department of Education and Learning, University of Applied Sciences and Arts of Southern Switzerland, Locarno, Switzerland; ^6^Department of Humanities, University of Naples Federico II, Naples, Italy

**Keywords:** Agenda 2030, wellbeing, health, inequalities, sustainability, quality education

The United Nations 2030 Agenda has defined 17 goals to promote sustainable development. Many of the goals can be connected to psychology or educational sciences, for example, improving health and wellbeing (SDG3), ensuring quality education (SDG4), promoting gender equality (SDG5) and decent work (SDG8), and reducing inequality (SDG10). The achievement of these goals requires the willingness at different levels (social, political, economic, legal, and psychological) to promote pathways toward sustainable scenarios, which go through the modification of the current development trends. In this regard, it becomes essential to increase the awareness and reflection of people on the issues that are discussed within the 2030 Agenda.

The research by Di Fabio et al. delves into the importance of building positive and supportive relationships as a fundamental aspect for healthy workers and organizations to cope with the current challenging work scenario. The results of this research underlined the value of positive relational management in relation to human capital sustainability leadership in organizations. In addition, connectedness and respect emerged as dimensions of positive relational management primarily associated with human capital sustainability leadership, and the authors suggest that these two dimensions could be assessed with greater attention in research and interventions aimed at studying and fostering positive relational management to promote human capital sustainability leadership.

Yousefi Afrashteh and Janjani focused on evaluating the mental state of Iranian adolescents between 11 and 18 years of age. Specifically, this research examined the psychometric properties of the Persian version of the MHC-SF (Keyes et al., 2008). The authors established that the MHC-SF is a psychometrically valid questionnaire for general mental wellbeing.

Di Fabio et al. conducted a study on 284 Italian university students, drawing on the perspective of the psychology of sustainability and sustainable development (PSSD) (Di Fabio and Rosen, 2018, 2020), a current area of research that supports the sustainability culture. This study examined the associations between acceptance of change and wellbeing, controlling for the effects of personality traits. The results obtained by the authors (acceptance of change explained additional variance over personality traits regarding hedonic and eudaimonic wellbeing) contribute to opening new perspectives for intervention; to improve wellbeing, individuals can be supported to effectively deal with the mutable environments of the current century.

Camussi et al., in their article, involved 835 Italian women and investigated the attitudes about roles, responsibilities, and expectations related to motherhood, as well as potential barriers people may encounter in their careers and values. Results show (1) a significant difference between the number of children women plan and the ideal number of children they would like; and (2) the parenthood choice is connected to the perception of social and gender inequity. Adopting a life design perspective (Savickas et al., 2009), the authors highlight the need to implement policies to support the birth rate; it is necessary to remove the obstacles to allow people to realize their family and life plans, supporting Goal 5, “Gender Equality”, and Goal 8, “Decent Work and Economic Growth”, of the 2030 Agenda.

Creed et al. analyzed the situation faced by many young people, which is defined as integrating a dual identity of working and studying. This research focuses on understanding the factors that contribute to managing work and study well. The analysis identified four profiles: (a) “balanced”, (b) “high work congruence and flexibility”, (c) “low work congruence and flexibility”, and (d) “low study congruence”. The authors demonstrated the value of deploying a person-centered approach, suggesting that different groups will also require different types of support and intervention.

Cantos-Egea et al. demonstrated that people in a situation of social exclusion tend to accumulate risk factors; they are related to having fewer psychosocial and cognitive resources to cope with stressful situations. Furthermore, in the absence of social integration and purpose in life, self-perceived health statuses decline. The authors underline that reducing inequality (SDG10) can also contribute to achieving other objectives of the 2030 Agenda, such as improving health and wellbeing (SDG3), ensuring quality education (SDG4), and promoting gender equality (SDG5) and decent work (SDG8).

Sun explored the relationship between self-objectification and career aspirations among young women from the perspective of objectification theory (Fredrickson and Robets, 1997). She focused on Chinese undergraduate women ranging in age from 16 to 21 years. Results showed that self-objectification was negatively correlated with self-esteem, career decision-making self-efficacy, and career aspirations. Self-esteem and career decision-making self-efficacy, both independently and serially, mediated the association between self-objectification and career aspirations. These findings, Sun argues, provide a better understanding of the negative consequences of self-objectification on career aspirations.

The contribution of Leão et al. demonstrated the positive effects of nature on human health through the lens of Complex Adaptive Systems. His contribution is particularly relevant insofar as theoretical models in this area are poorly developed, and they construct their own model: “A time with e-Natureza” (e-Nature). This model not only looks at human wellbeing but also supports nature conservation and is based on four types of experiences: (1) esthetic and emotional experience, (2) multisensory integration experience, (3) knowledge experience, and (4) engagement experience. This integrated approach recognizes the mutual benefits of human–nature interaction and offers valuable insights for future research and practical applications in the fields of nature and health.

The study by Gramaxo et al. involved 2.708 participants with the aim of uncovering Portuguese students' perspectives concerning a happy school. To the question “What makes you happy in school?”, the sample provided answers that underlined the importance of relationships with friends and teachers and teachers' attitudes, competencies, and capacities as elements of a happy school. On the contrary, excessive workload and bullying were at the root of school unhappiness. This study shows interesting results in understanding the happiness dimension of the Portuguese student population and is consistent with objective 4 of the 2030 Agenda, the promotion of quality education.

The study by Wang et al. was conducted on 1.172 primary school teachers. Through a survey research methodology, the authors found that technology use intensity directly impacted work–family conflicts and personal health and indirectly impacted them via the agency effects of Technostress. Furthermore, school support moderates the indirect relationship among technology intensity, work–family conflicts, and health issues. The findings of this study highlight the importance of promoting teacher wellbeing, which is consistent with Goal 3 of the 2030 Agenda regarding the promotion of health and wellbeing.

Espinoza-Díaz et al. analyzed the psychological wellbeing of teachers in relation to their professional performance. The aim of the study was to assess the influence of personality factors, emotional intelligence, burnout, and the psychosocial climate derived from the teachers' work environment on their levels of psychological wellbeing. The authors show that the main predictor of burnout in teachers is the perception of disorganization in the work environment. However, if emotional stability is high, this tends to be less affected.

Finally, Siqi and Hong examined the relationships among teaching-research conflict, career adaptability, justice climate, job burnout, and turnover intention. The research involved 858 Chinese university teachers. Results showed that job burnout mediates the relationship between teaching-research conflict and turnover intention; career adaptability plays a moderating role in the connection between job burnout and turnover intention; justice climate exhibits a cross-level interaction effect concerning the relationship between teaching-research conflict and turnover intention. These results show evidence for the urgency of fostering equitable environments in higher education.

Studies have shown that, among researchers, there is a particular concern about one of the goals of the 2030 Agenda: improving mental health and wellbeing. Research results have pointed to the systemic nature of wellbeing, considering personal as well as contextual and environmental factors. Research in the field of psychology and educational sciences must stimulate the study and understanding of the dynamics that also underlie the other goals of the 2030 Agenda. Universities can contribute to achieving the goals of the 2030 Agenda by promoting sustainable behaviors and increasing the number of field research. The studies collected in this Research Topic provide a basis for researchers in psychology and related disciplines who can help to reflect on sustainable development goals.

## Author contributions

AZ: Writing—original draft, Writing—review & editing. CM-M: Writing—original draft, Writing—review & editing. SR-R: Writing—original draft, Writing—review & editing. PM: Writing—original draft, Writing—review & editing. JM: Writing—original draft, Writing—review & editing. AP: Writing—original draft, Writing—review & editing.
